# The effects of age and sex on the incidence of multiple step saccades and corrective saccades

**DOI:** 10.3389/fnagi.2022.963557

**Published:** 2022-09-07

**Authors:** Wenbo Ma, Mingsha Zhang

**Affiliations:** State Key Laboratory of Cognitive Neuroscience and Learning and International Data Group (IDG)/McGovern Institute for Brain Research, Division of Psychology, Beijing Normal University, Beijing, China

**Keywords:** male, female, senescence, vertical saccades, horizontal saccades, reactive saccades, saccade, multiple step saccades

## Abstract

**Objective:**

Although multiple step saccades (MSS) is occasionally observed in healthy subjects, it is more pronounced in patients with aging-related neurodegenerative diseases, particularly Parkinson’s disease (PD). Thus, MSS has been treated as a complementary biomarker for diagnosing PD. Despite the aforementioned knowledge, several questions remain unexplored: (1) How does aging affect MSS? (2) Is there a sex difference in MSS? (3) Are there differences in MSS between vertical and horizontal saccades? (4) Are MSS and corrective saccade (CS) the same behavior? (5) How do age and sex affect CS? The objectives of the present study are to address these questions.

**Method:**

Four hundred eighty healthy participants were recruited to perform a visually guided reactive saccade task. Participants were divided into six groups according to their ages. Each group consisted of 40 male and 40 female participants. Eye movements were recorded with infrared eye trackers.

**Results:**

The incidence of MSS increased as a function of age, whereas the incidence of CS first increased with age 20–49 and then decreased with age 50–79. The incidences of both MSS and CS did not show sex differences. The incidence of MSS in vertical saccades was significantly higher than that in horizontal saccades, and their difference increased with increasing age, whereas the incidence of CS showed a reversed pattern.

**Conclusion:**

Age and saccadic direction affect the occurrences of MSS and CS differently, indicating that MSS and CS are different saccadic behaviors. In addition, measuring saccades could reliably reflect the function of human’s brain which is affected by aging.

## Introduction

Saccades are rapid and congruent jumps of the eyes that direct the fovea of the retina onto various objects of interest. Commonly, a saccade consists of a primary saccade that covers all or most of the distance between the fixation point and the target location, which might be followed shortly by a small-amplitude saccade (corrective saccade, CS) if needed. However, eyes do not always jump with the common form, and sometimes they engage in a series of at least two smaller amplitude (hypometric) saccades, namely, multiple step saccades (MSS) (Troost et al., 1974). Although MSS has been observed in healthy children, adults and elderly individuals (Kimmig et al., 2002; Van Donkelaar et al., 2007), it has been clearly more pronounced in patients with brain diseases (Troost et al., 1974), particularly in patients with Parkinson’s disease (PD) (Jones and DeJong, 1971; Corin et al., 1972; Troost et al., 1974; Teräväinen and Calne, 1980; White et al., 1983; Hotson et al., 1986; Lueck et al., 1990, 1992; Van Gisbergen et al., 1992; Kimmig et al., 2002). Thus, some investigators have argued that the MSS could serve as a behavioral biomarker for the diagnosis of PD (Blekher et al., 2009; Ma et al., 2022). Since PD is the second most common neurodegenerative disease, understanding the general effect of aging on the incidence of MSS will provide important information for understanding the mechanisms of MSS in PD. Surprisingly, while seeking the relevant literature, we only found two papers that studied the effect of age on the incidence of MSS (Van Donkelaar et al., 2007; Litvinova et al., 2011). While one study reported that the incidence of MSS gradually decreased following the development of the brain from childhood to young adulthood (Van Donkelaar et al., 2007), another study reported that the incidence of MSS increased significantly after the age of 60 (Litvinova et al., 2011). However, there are some limitations in the second study due to the small number of healthy participants and the inclusion of CS as a part of MSS (see the following second paragraph for detail). Therefore, the first objective of this study is to systematically assess how aging affects the incidence of MSS with a large sample of participants.

Furthermore, the prevalence of PD is 1.5–2.0 times higher in males than in females (Van Den Eeden et al., 2003). Thus, it is important to know whether there is a sex difference in the incidence of MSS. Surprisingly, again, we failed to find a study that had approached this question. Therefore, the second objective of this study is to address whether sex affects the incidence of MSS.

Although CS shares certain features with MSS, e.g., both are small-amplitude saccades, CS is frequently observed in healthy participants (Cohen and Ross, 1978; White et al., 1983) and is treated as a physiological behavior (Troost et al., 1974). In contrast, MSS is clearly more pronounced in PD patients (Jones and DeJong, 1971; Corin et al., 1972; Troost et al., 1974; Teräväinen and Calne, 1980; White et al., 1983; Hotson et al., 1986; Lueck et al., 1990, 1992; Van Gisbergen et al., 1992; Kimmig et al., 2002) and is assumed to be a pathological behavior (Troost et al., 1974). Thus, the third objective of this study is to distinguish CS from MSS and explore whether and how age and sex affect the incidence of CS.

Finally, it is well known that vertical and horizontal saccades are controlled by different neural circuits (Lemos et al., 2016; Irving and Lillakas, 2019) and that neurodegenerative diseases, e.g., PD and progressive supranuclear palsy, primarily damage the performance of saccades in the vertical direction (Bhidayasiri et al., 2001; Antoniades and Kennard, 2015; Lemos et al., 2016; Jung and Kim, 2019). However, it is not clear whether there is a difference regarding the incidences of MSS and CS between vertical and horizontal saccades. Thus, the fourth objective of this study is to address this question.

## Materials and methods

### Participants

Four hundred eighty neurologically and psychologically healthy participants were recruited for the present study. In the present study, we recruited the participants in the college and residential community. Participants were divided into six groups according to their ages. Each group (10 years range) consisted of 40 male and 40 female participants (Table 1). The sample size was ascertained by G-power software, effect size 0.6, α 0.05, β 0.1 and power is 1-β, 0.9. The G-Power was designed as a general stand-alone power analysis program for statistical tests commonly used in social and behavioral research (Faul et al., 2007). Moreover, to ensure that the participants had no cognitive impairments, each participant completed the Folstein mini-mental state examination (MMSE) with a score ≥26. It was certain that all participants did not have motor complaint or exhibit symptoms of PD. All participants had normal or corrected-to-normal vision. All participants were informed about the study’s objectives and provided written consent to take part in the study. The experimental protocol was approved by the Ethics Committee of Beijing Normal University.

**TABLE 1:** Demographic and clinical characteristics of the subjects.

Groups	*N* (male/female)	Age^a^ (male)	Age^a^ (female)	MMSE^a^ (male)	MMSE^a^ (female)
1	40/40	24.85 ± 3.00	24.83 ± 2.50	29.31 ± 0.88	29.09 ± 0.95
2	40/40	32.58 ± 2.27	34.53 ± 2.78	28.92 ± 0.92	28.89 ± 0.89
3	40/40	44.58 ± 3.06	44.75 ± 2.91	28.79 ± 0.99	28.82 ± 1.13
4	40/40	55.15 ± 2.83	55.38 ± 2.30	28.89 ± 0.98	28.78 ± 0.98
5	40/40	65.53 ± 2.73	64.65 ± 2.39	28.72 ± 0.90	28.61 ± 0.95
6	40/40	73.08 ± 2.48	72.85 ± 2.30	28.31 ± 0.84	28.57 ± 0.93

^*a*^Mean ± SD.

### Experimental task

We employed a visually guided reactive saccade task in the present study for two major reasons: first, it is one of the simplest saccadic tasks to be performed; second, the definition and criteria to classify MSS and CS are clearer in this task. Each block consisted of 40 trials.

**FIGURE 1:**
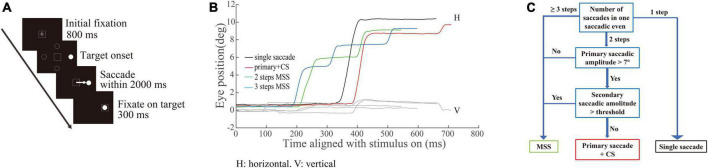
Schematic illustration of saccadic tasks and methods of quantifying saccades. **(A)** Paradigm of the visually guided reactive saccade task: white crosses and circles represent fixation points and targets, respectively. Dashed squares indicate the location of fixation. Dashed circles indicate other potential locations of the target. The white arrow represents the required saccade. **(B)** Exemplified eye trace of an elderly subject. The *X*-axis represents the time aligned with the saccadic target onset. The *Y*-axis represents the eye position. Different colors denote different types of saccadic events. **(C)** Illustration classifying the different types of saccadic events.

*Visually guided reactive saccade task* (Figure 1A). Each trial began with a white cross (fixation point) appearing at the center of the screen for 800 ms. The participant was required to fix at the fixation point (check window 4° in radius) for 300 ms, and then the fixation point disappeared. Simultaneously, a white dot (target) randomly appeared in one of four peripheral locations (right, left, up, and down, with an eccentricity of 10°). The participants were instructed to make a saccade toward the target as accurately and quickly as possible. The target disappeared only after the eye entered and was maintained in the check window (4° in radius) for 300 ms. The size of the fixation points and target were 1° in length or diameter, respectively. A blank screen was interposed between trials with an interval of 800 ms. Each trial lasted only 3–4 s, so each participant in the study spent approximately 3 min on this test.

### Data acquisition

Eye movements were monitored at 1 kHz with a head-restrained infrared video-based eye tracker (EM-2000R, Jasmine Science and Technology Ltd., Beijing, China; Eye Link 1000 desktop mount, SR Research, Ltd., ON, Canada). Participants were seated in a dark room 57 cm away from the monitor (XL2720-B; resolution: 1,920 × 1,080; 27-inch; refresh rate: 100 Hz). The system was calibrated prior to the experiment by having the participants make saccades to nine targeting locations, which were distributed symmetrically around the center of screen. The background luminance of the monitor was 0.08 cd/m^2^, and the luminance of visual stimuli was 23.9 cd/m^2^. Stimuli presentation and behavioral data collection were controlled by MATLAB (R2009b; MathWorks, Natick, MA, United States) with Psychtoolbox (PTB-3) running on a Windows system PC (HP).

### Quantitative measures of saccades

The detailed methods for the quantitative measurements of saccades have been reported recently in one of our paper (Ma et al., 2022). Here, we just give a brief introduction as following. A velocity threshold was set to find all responsive saccades from target onset to the end of the trial. The velocity threshold was the mean velocity +2.58 × standard deviation (STD) (99% confidence interval) during a time interval of 200 ms prior to the target onset. Each responsive saccade was defined as its velocity was greater than the velocity threshold and its duration was greater than 10 ms. The intersaccadic interval was the time from the end time of the preceding saccade to the start time of the current saccade. One criterion for defining the MSS is that the intersaccadic interval is larger than one minimum value and smaller than one maximum value. The minimum and maximum values were obtained from the distribution of the intersaccadic intervals. Since the distribution of intersaccadic intervals among the six groups are different, the minimum and maximum values were varied, i.e., the range of the intersaccadic intervals of the six groups were 30–215, 32–224, 31–304, 31–353, 31–305, and 33–363 ms, respectively. The first responsive saccade was defined as a saccade with a minimum amplitude of 2° and a minimum latency of 30 ms, and its direction was toward the target location. While we plotted the eye traces, we found that there were different types of saccades with varied spatiotemporal properties (Figure 1B). It is obvious that there are different types of saccadic trajectories including the typical saccades (a single saccade or a saccade with large gain followed by a CS, black and red traces) and the MSS with 2 or 3 saccades with small gains (green and blue traces). To classify the different types of saccades well, we defined different types of saccades mainly based on the number of saccades as shown in Figure 1C (for more detailed information, refer to Ma et al., 2022).

In addition, since the total number of trials in each session was 40, to ensure that there was a sufficient number of correct trials for data analysis, the incidences of MSS and CS were calculated when the correct rate of a session was ≥70%. The proportions of the trials that met our criteria were 93.57, 89.87, 92.26, 91.82, 91.69, and 91.22% for the six groups of participants, respectively. In addition, the incidences of MSS and CS in horizontal and vertical saccades were calculated when the correct rates of the two directions were ≥the mean −1.5 × STD (minimal trial number was 10) of each group of participants.

### Statistical analysis

The Kruskal–Wallis test (a non-parametric approach to one-way ANOVA) was applied to determine the significant difference among six independent age groups of participants based on the incidence of MSS and CS. This was corrected by the Bonferroni correction, and α was set to 0.05. If there were significant differences among the six groups of participants, a *post hoc* test was performed to determine the significance between each pair of participants either by the Wilcoxon rank-sum test for unpaired data or by the Wilcoxon signed-rank test for paired data. The α was set to 0.05.

Furthermore, we employed a curve fitting tool (MATLAB, cftool function) to examine the relationship between age and the incidence of MSS and CS. The fitting function between age and the incidence of MSS was f(*x*) = *a*_1_*x* + *a*_2_, where *x* denotes the age of the participants, *a*_*1*_ is the coefficient of *x, a*_*2*_ is a constant and f(*x*) denotes the incidence of MSS. Moreover, this fitting function was also applied to examine the relationship between the age of participants and the incidence difference of MSS or CS in vertical saccades and MSS or CS in horizontal saccades. For the correlation between age and the incidence of CS, we employed two linear fitting curves to fit our data from the age range of 20–49 years and the age range of 49–79 years. To exclude the possibility that the decreased incidence of CS in the elderly participants is due to their spatially accurate performance of primary saccades, i.e., the reduced spatial error between the endpoints of primary saccades and the location of the target, we calculated the averaged spatial errors of each group of participants and made a direct comparison between the incidences of CS and the spatial errors of primary saccades. In addition, we justified the goodness of fit curves based on the statistical results of the fit function, including the sum of squares due to error (SSE), the root mean squared error (RMSE), the coefficient of determination (*R*-square) and the degrees-of-freedom adjusted coefficient of determination (adjusted *R*-square). A more ideal fitting curve has a lower SSE and RMSE and a larger *R*-square and adjusted *R*-square.

## Results

### The incidences of multiple step saccades and corrective saccade are similar between male and female participants

**FIGURE 2:**
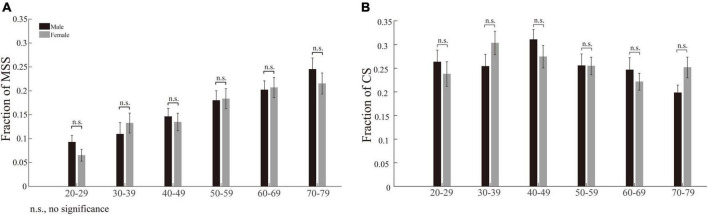
Incidences of MSS and CS between male and female participants among six age groups. **(A)** The incidence of MSS between male and female participants. There was no significant difference in MSS incidence between males and females among all age groups. **(B)** The incidence of CS between male and female participants. There was no significant difference in CS incidence between males and females among all age groups. Error bars show the SEM; n.s., no significant difference (Wilcoxon rank-sum test).

To assess whether sex affects the incidences of MSS and CS, we compared the incidences of MSS and CS between male and female participants with similar ages. The results show that the incidences of MSS and CS were not significantly different between males and females, from young to elderly participants (Figure 2, *p* > 0.05 for all comparisons, Wilcoxon rank-sum test). Such results demonstrate that sex does not affect the incidence of MSS and CS. Therefore, we pooled the data of MSS and CS from male and female participants within one group together for further analysis.

### The incidences of multiple step saccades and corrective saccade correlate with age differently

**FIGURE 3:**
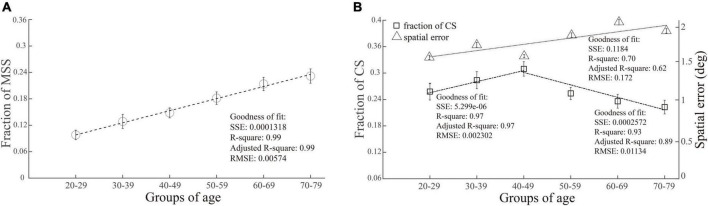
Correlation between the incidences of MSS and CS with age. The incidence of MSS was positively correlated with age, as shown in (**A**), while the incidence of CS first increased and then decreased with age, as shown in (**B**) (squares and dashed line). The spatial errors of primary saccades (distance between the endpoints of primary saccades and target location) increased with increasing age (**B**, triangle and dotted line). SSE, the sum of squares due to error; RMSE, root mean squared error; *R*-square, coefficient of determination; adjusted *R*-square, degrees-of-freedom adjusted coefficient of determination.

While we plotted the incidence of MSS as a function of age, the incidence of MSS gradually increased with increasing age (Figure 3A). In contrast, the incidences of CS first increased from age 20 to 49 and then decreased from age 50 to 79 (Figure 3B, squares and dashed lines). Do elderly participants make more spatially accurate primary saccades, i.e., are the endpoints of primary saccades closer to the location of the target, than young participants, which causes the decreased incidence of CS in elderly participants? To address this question, we plotted the averaged distance between the endpoints of primary saccades and the location of the target (spatial error) as a function of age, as shown in Figure 3B (triangle and dotted line). It is clear that the spatial errors increase with increasing age. Such results indicate that the capability to correct the spatial error of saccade declines in the elderly participants.

The different correlation patterns between MSS and CS with age indicate that, although both of them are small-amplitude saccades, they are different saccadic behaviors and might be generated by different neural mechanisms.

### The incidences of multiple step saccades are higher in vertical saccades than in horizontal saccades, and the differences between them become larger with increasing age

**FIGURE 4:**
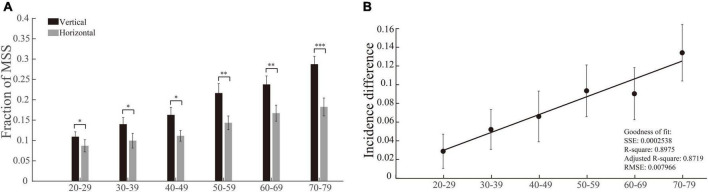
Incidences of MSS in vertical and horizontal saccades and their difference as a function of age. **(A)** The incidence of MSS in vertical and horizontal saccades. The incidence difference of MSS between vertical and horizontal saccades was significantly different in all age groups. **(B)** The incidence difference of MSS between vertical and horizontal saccades as a function of age. The incidence difference of MSS between vertical and horizontal saccades was positively correlated with age. Error bars show the SEM; *p < 0.05, ^**^p < 0.01, ^***^p < 0.001, n.s., no significant difference (Wilcoxon rank-sum and sign-rank tests). SSE, the sum of squares due to error; RMSE, root mean squared error; *R*-square, coefficient of determination; adjusted *R*-square, degrees-of-freedom adjusted coefficient of determination.

Considering the fact that the vertical and horizontal saccades are generated by different brain structures and circuits (Lemos et al., 2016; Irving and Lillakas, 2019), we compared the incidences of MSS between vertical and horizontal saccades in each group of participants. By performing this analysis, we intend to address the following two questions: (1) Are the incidences of MSS different between vertical and horizontal saccades? (2) If there is a difference, how does age affect such a difference? Although the incidences of MSS gradually increased in both vertical and horizontal saccades with increasing age (*p* = 0.0004 and *p* = 4.5 × 10^–9^ for horizontal and vertical comparisons, Figure 4A, Kruskal–Wallis test), there was a clear trend that the incidences of MSS in vertical saccades were higher than those in horizontal saccades across all groups of participants with different ages (Figure 4A, effect sizes of the significant groups are 0.13, 0.22, 0.21, 0.08, and 0.25). To further quantify the difference in the incidence of MSS between vertical and horizontal saccades, we subtracted the incidence of MSS in the horizontal saccades by the incidence of MSS in the vertical saccades. The incidence differences in MSS increased with increasing age and were positively linearly correlated with age (Figure 4B). Such results indicate that natural senescence causes more MSS in vertical saccades than in horizontal saccades.

### The incidences of corrective saccade in vertical saccades, but not in horizontal saccades, gradually decrease with increasing age

**FIGURE 5:**
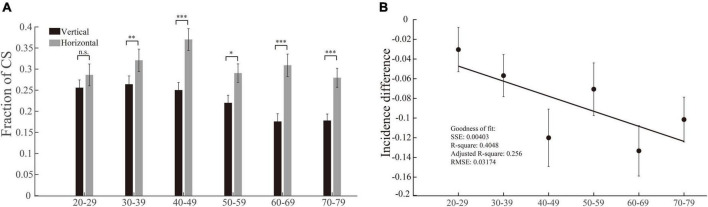
Incidences of CS in vertical and horizontal saccades and their difference as a function of age. **(A)** The incidence of CS in vertical and horizontal saccades. The incidence of CS in horizontal saccades was significantly higher than that in vertical saccades in all age groups except the 20–29 age group. **(B)** The incidence difference of CS between vertical and horizontal saccades as a function of age. The incidence differences of CS in vertical and horizontal saccades and age were negatively correlated with age. Error bars show the SEM; *p < 0.05, ^**^p < 0.01, ^***^p < 0.001, n.s., no significant difference (Wilcoxon rank-sum and sign-rank tests). SSE, the sum of squares due to error; RMSE, root mean squared error; *R*-square, coefficient of determination; adjusted *R*-square, degrees-of-freedom adjusted coefficient of determination.

We used the same method and logic (same as in section “The incidences of multiple step saccades are higher in vertical saccades than in horizontal saccades, and the differences between them become larger with increasing age”) to perform the data analysis of CS. While we compared the incidences of CS between vertical and horizontal saccades in each group of participants, the most notable result was that the incidences of CS in the vertical saccades gradually decreased with increasing age (*p* = 0.0032, Kruskal–Wallis test), whereas the incidences of CS in the horizontal saccades remained at a similar level (*p* = 0.1415, Kruskal–Wallis test). In addition, there was a clear trend that the incidences of CS were lower in vertical saccades than in horizontal saccades across all groups of participants with different ages (Figure 5A, effect sizes of the significant groups are 0.11, 0.18, 0.36, 0.23, 0.36, and 0.30). Thus, the incidence differences of CS between vertical and horizontal saccades became larger with increasing age, and they were negatively linearly correlated with age (Figure 5B). Such results indicate that natural senescence causes a gradual decrease in CS in vertical saccades but not in horizontal saccades, which is opposite to the aging effect on MSS, as shown in Figure 4. Therefore, such results demonstrate again that the MSS and CS might be induced by different neural mechanisms.

## Discussion

In the present study, we explored the effects of natural senescence and sex on the incidences of MSS and CS in a visually guided reactive saccade task. MSS and CS have been treated as pathological and physiological behaviors (Troost et al., 1974). Since aging can cause neurodegeneration in the central nervous system, we thus hypothesize that the incidences of MSS and CS may have different correlation patterns with age. Since the vertical and horizontal saccades are generated by different neural circuits (Lemos et al., 2016; Irving and Lillakas, 2019), we thus hypothesize that the incidences of MSS and CS may be different between vertical and horizontal saccades. Furthermore, since more recent studies reported that there is no significant difference in the performance of visually guided reactive saccades between male and female participants (Fujiwara et al., 2000; Bonnet et al., 2013), we assume that the incidences of MSS and CS may be not significantly different between males and females. To test these hypotheses, we recruited a large sample of participants and compared the incidences of MSS and CS among different ages and between males and females. Our results showed that (1) the incidences of MSS and CS were not significantly different between males and females (Figure 2); (2) the incidence of MSS was positively correlated with age (Figure 3A), whereas the incidence of CS was positively correlated with age in the range of 20–49 and negatively correlated with age in the range of 50–79 (Figure 3B); and (3) the incidence of MSS in vertical saccades was significantly higher than in horizontal saccades (Figure 4), whereas the incidence of CS in vertical saccades was significantly lower than in horizontal saccades (Figure 5). Therefore, these results support the aforementioned hypotheses.

### Multiple step saccades and corrective saccade are different saccadic behaviors

In previous studies whether CS is a part of MSS was a matter of controversy. While some studies treated CS and MSS as different behaviors and excluded CS from the analysis of MSS (Troost et al., 1974; Bötzel et al., 1993; Van Donkelaar et al., 2007), others considered that CS was a part of MSS and included CS in the analysis of MSS (Becker and Fuchs, 1969; Oliva, 2001). Such a difference in data analysis might be a critical reason for the inconsistencies among previous MSS studies. For instance, some studies reported that the incidence of MSS in visually guided reactive saccades was higher in PD patients than in healthy controls (Jones and DeJong, 1971; Corin et al., 1972; White et al., 1983), whereas other studies reported no significant difference between PD and healthy subjects (Crawford et al., 1989; Lueck et al., 1990, 1992; Van Gisbergen et al., 1992; Kimmig et al., 2002; Blekher et al., 2009). In the present study, we excluded CS from the analysis of MSS and analyzed whether and how the age affected the incidence of each of them. Our results clearly showed that the effects of age on the incidences of MSS and CS were very different (Figures 3–5). Such findings support the argument that MSS and CS are distinct behaviors (Troost et al., 1974).

### The effects of age on the incidences of multiple step saccades and corrective saccade are different

It is well known that age is an important factor affecting the performance of saccades (Munoz et al., 1998; Yang and Kapoula, 2006, 2008; Peltsch et al., 2011; Seferlis et al., 2015). For instance, aging could extend the saccadic reaction time, slow the saccadic peak velocity and decrease the occurrence of express saccades. Since the incidence of MSS increases significantly in PD patients, it has been argued that MSS could serve as a complementary behavior biomarker for the diagnosis of PD (Blekher et al., 2009; Ma et al., 2022). Moreover, since PD is the second most common neurodegenerative disease, we assume that studying the effect of natural senescence on the incidence of MSS might shed light on understanding the neuronal mechanism underlying the generation of MSS in PD.

While one study reported that the incidence of MSS increased significantly after the age of 60 (Litvinova et al., 2011), our results show that the incidence of MSS was positively and linearly correlated with age from 20 to 79 years old (Figure 3A), indicating that the incidence of MSS increases following natural degeneration. Conversely, a negative correlation was found between the incidence of MSS and age from childhood to young adulthood (Van Donkelaar et al., 2007), indicating that the incidence of MSS decreases following natural development. Taking the results of these two studies together, we speculate that the relationship between the incidence of MSS and age appears to have an asymmetric “U” shape. We attribute such an asymmetric “U” shape to two separate processes: a developmental process from childhood to young adulthood and a natural degeneration process from adulthood to older age.

In contrast to MSS, the effect of age on the incidence of CS showed a very different pattern. The incidence of CS increased with increasing age in the range of 20–49 and then decreased with increasing age in the range of 50–79 (Figure 3B, squares and dashed line). We further analyzed the relationship between the spatial errors of primary saccades (see section “Quantitative measures of saccades”). The results showed that spatial errors increased with increasing age (Figure 3B, triangle and dotted line). Such results indicate that the capability to correct the spatial error of saccade declines in the elderly.

### The possible mechanisms underlying the effects of age on the incidences of multiple step saccades and corrective saccade

Why does age affect MSS and CS differently? To answer this question, we need to understand the natural characteristics of MSS and CS first. Although it has been argued that MSS and CS reflect pathological and physiological behaviors, respectively (Troost et al., 1974), the principal functions of MSS and CS are the same, i.e., to correct the spatial errors of primary saccades (Weber and Daroff, 1972; Troost et al., 1974; Kimmig et al., 2002). Therefore, some researchers treated MSS as multiple CSs (Becker and Fuchs, 1969; Oliva, 2001). However, based on the definitions used in the present study and other studies (Kimmig et al., 2002; Van Donkelaar et al., 2007; Ma et al., 2022), the remarkable difference between MSS and CS is that there is one CS in a CS trial, whereas there are multiple CSs in a MSS trial. According to the models of the saccadic eye movement control that was originally proposed by Robinson (1973), we think that the generation of MSS and CS might share the similar mechanism, i.e., the existence of error signal between the saccadic endpoint and the saccadic goal triggers small amplitude saccade(s) to make accurate/spatial correction. Such assumption is supported by studies that have found both frontal eye field (FEF) and cerebellum are involved in the generation of MSS and CS (Optican and Robinson, 1980; Murthy et al., 2007; van Donkelaar et al., 2009). Thus, the number of CSs in a trial is highly relied on the gain of primary saccade, i.e., the larger of the primary saccadic gain, the smaller number of CSs or not at all.

It is well known that aging causes numerous changes in the brain, such as neuron loss, dendrite loss, reduced branching, and altered transmitter metabolism (Buckner et al., 2000; Head et al., 2004; Kramer et al., 2007). These changes can be observed in cortical (e.g., prefrontal and parietal cortex) and subcortical regions (e.g., basal ganglia, cerebellum, and superior colliculus) that play critical roles in the control of saccades (Schiller et al., 1979; Gaymard et al., 1998, 2003; Schiller and Tehovnik, 2005). The decline of the function of saccadic control system will cause various deficits in saccades, in particular in the reduction of saccadic gain (Versino et al., 1992; Fahle and Wegner, 2000; Sharpe and Zackon, 1987; Irving and Lillakas, 2019). As a result, the incidence of MSS increases and incidence of CS decreases in natural senescence.

### The incidences of multiple step saccades and corrective saccade are different between vertical and horizontal saccades

One noticeable observation in the present study is that the incidences of MSS are significantly higher in vertical saccades than in horizontal saccades across six groups of subjects, from young to elderly (Figure 4A). Furthermore, the incidence difference of MSS between vertical and horizontal saccades is positively and linearly correlated with the increase of age (Figure 4B). In contrast, the reversed results are seen in CS (Figure 5). What might cause such difference?

It is well known that the vertical and horizontal saccades are controlled by different brain regions and neural networks, e.g., the caudal pons is important in the control of horizontal saccades, whereas the rostral mesencephalon is important in the control of vertical saccades (Leigh et al., 2007; Bonnet et al., 2013; Lemos et al., 2016; Irving and Lillakas, 2019). While little attention has been given in the literature to the cortical control of vertical saccades, they seem to require bilateral cortical activation of the oculomotor network to be executed, whereas horizontal saccades are generated by a predominantly contralateral activation of the same underlying network (Lemos et al., 2016). Comparing vertical saccades with horizontal saccades, a higher level activation is evoked in the right FEF, the posterior lobe of cerebellum and the superior temporal gyrus (Lemos et al., 2016). The stronger cortical activation in vertical saccades indicates that the more complicated neural responses are required in control of vertical saccades than that in horizontal saccades. The requirement of cooperation of bilateral hemispheres and more complicated neural activity in control of vertical saccades implies the higher probability to create errors. Supportively, it has been found that the vertical saccades were more hypometric than their horizontal counterparts across all ages (Irving and Lillakas, 2019), and aging primarily affects the performance of vertical saccades, particularly reducing the saccadic gain, rather than of horizontal saccades (Chamberlain, 1971; Jenkyn et al., 1985; Irving and Lillakas, 2019). Thus, it is more likely to make multiple CSs, i.e., MSS in the present study, in vertical saccades than in horizontal saccades, whereas to make single CS, i.e., CS in the present study, in horizontal saccades than in vertical saccades.

### Sex affects the incidence of multiple step saccades and corrective saccade similarly

Some previous studies demonstrated that sex could cause differences in saccadic performance, such as a shorter latency in female participants in visually guided saccade tasks and different saccade amplitudes and durations between male and female participants in free viewing tasks (Scientific et al., 1983; Nagel-leiby et al., 1990; Abdi Sargezeh et al., 2019). However, more recent studies demonstrated no significant difference in saccadic performance between males and females (Wilson et al., 1993; Fujiwara et al., 2000; Bonnet et al., 2013). In the present study, we found no significant difference between male and female participants in the incidences of MSS and CS in the visually guided reactive saccade task (Figure 2). Such results are consistent with the findings of previous studies that demonstrate no sex difference in performing visually guided saccade tasks (Wilson et al., 1993; Fujiwara et al., 2000; Bonnet et al., 2013). Therefore, the similarity in the incidence of MSS between male and female participants indicates that employing MSS as a behavioral marker for the diagnosis of PD is not affected by sex.

## Limitations of the study

Since the present study is a psychophysical experiment, we did not directly assess the relationship between neural activity and the incidence of MSS and CS. We plan to simultaneously measure/interrupt brain activity while subjects perform saccadic tasks in future studies to directly explore the neural mechanisms underlying the generation of MSS and CS.

## Conclusion

Age and saccadic direction affect the occurrences of MSS and CS differently, indicating that MSS and CS are different saccadic behaviors and are probably generated by different neural mechanisms. In addition, measuring saccades could reliably reflect the function of human’s brain which is affected by aging.

## Data availability statement

The raw data supporting the conclusions of this article will be made available by the authors, without undue reservation.

## Ethics statement

The studies involving human participants were reviewed and approved by the Ethics Committee of Beijing Normal University. The patients/participants provided their written informed consent to participate in this study.

## Author contributions

WM and MZ designed the experimental paradigms. WM performed the experiments, analyzed the data, and wrote the manuscript. MZ supervised the experiments and wrote the manuscript. Both authors contributed to the article and approved the submitted version.
